# Development of the SCI-BodyMap—Measuring Mental Body Representations in Adults With Spinal Cord Injury: Protocol for Item Generation, Reliability, and Validity Testing

**DOI:** 10.2196/72370

**Published:** 2025-09-08

**Authors:** Sydney Carpentier, Sara Bottale, Nicole Cenci, Mauro Cracchiolo, Daniele De Patre, Julian Pablo Gorosito, Ilaria Grimaldi, Mara Melo, Bianca Polinelli, Marco Rigoni, Fortunata Romeo, Marina Zernitz, Ann Van de Winckel

**Affiliations:** 1 Rehabilitation Science Graduate Program, Department of Family Medicine and Community Health Medical School University of Minnesota-Twin Cities Minneapolis, MN United States; 2 Centro Studi di Riabilitazione Neurocognitiva Villa Miari - Villa Miari (Study Center for Cognitive Multisensory Rehabilitation) Santorso, Vicenza Italy; 3 Instituto Avencer Pinheiros - São Paulo Brazil; 4 NeuroRiab – Specialist Center for Post-Stroke and Neurological Rehabilitation Teramo Italy; 5 Istituto Universitario di Studi Superiori Pavia Italy; 6 Division of Physical Therapy and Rehabilitation Science, Department of Family Medicine and Community Health Medical School University of Minnesota-Twin Cities Minneapolis, MN United States

**Keywords:** spinal cord injury, mental body representations, body awareness, visuospatial body map, reliability, validity

## Abstract

**Background:**

Approximately 69% of Americans with spinal cord injury (SCI) have neuropathic pain. Research suggests that impairments in mental body representations (MBRs; ie, representations of the body in the brain) likely contribute to neuropathic pain. Clinical trials in adults with SCI, focused on restoring MBR, led to improvements in sensation and movement as well as neuropathic pain relief. Scales measuring aspects of MBR exist, but none of them assess SCI-related MBR impairments.

**Objective:**

As our first aim, we will generate items for a new MBR scale for adults with SCI (the SCI-BodyMap). As our second aim, we will assess the interrater reliability, test-retest reliability, concurrent validity, face validity, and utility of the SCI-BodyMap.

**Methods:**

Our preliminary work will encompass initial item generation by SB, an Italian physical therapist (PT) specialized in cognitive multisensory rehabilitation, which is a therapeutic approach that focuses on restoring MBR in adults with neurological disorders and chronic pain. Further item refinements will be carried out by Italian PTs (n=7) and Brazilian PTs (n=3) specialized in cognitive multisensory rehabilitation. In aim 1, American PTs or occupational therapists (n=8) and adults with SCI (n=8) will provide feedback on the SCI-BodyMap. Next, American PTs or occupational therapists (n=3) will administer the SCI-BodyMap to adults with SCI (n=3) and provide more feedback during an in-person visit. In aim 2, four assessors will administer the SCI-BodyMap to adults with SCI (n=30) for interrater reliability. The self-report items will be administered at 2 separate time points to assess test-retest reliability. We will also administer the SCI-BodyMap to uninjured adults (n=30) to identify whether healthy adults score statistically different on the scale than adults with SCI. We will assess concurrent validity through correlations between the MBR scale, the Revised Body Awareness Rating Questionnaire, and the Multidimensional Assessment of Interoceptive Awareness-2.

**Results:**

As of August 2025, we have enrolled 8 PTs or occupational therapists and 8 adults with SCI for aim 1 as well as 29 adults with SCI and 13 uninjured adults for aim 2.

**Conclusions:**

A reliable and valid MBR scale is needed to identify MBR deficits and evaluate intervention effects on MBR outcomes in adults with SCI. Improving MBR can lead to safer, more efficient day-to-day activities (eg, transfers); promote functional independence and quality of life; reduce neuropathic pain and spasms; and improve sensorimotor function.

**International Registered Report Identifier (IRRID):**

DERR1-10.2196/72370

## Introduction

### Background

There are approximately 308,620 adults with spinal cord injury (SCI) in the United States, with approximately 18,421 new cases of SCI every year [1]. Pain is one of the most common secondary conditions of SCI and the most difficult to treat [2]. This is especially true for chronic neuropathic pain, present in 69% of adults with SCI [3]. Neuropathic pain is described as sharp, shooting, stabbing, electric, or burning and is sometimes experienced as excruciating pain at, below, or above the level of spinal injury [2,4].

When parts of the body are paralyzed and have impaired or absent sensation after an SCI, the brain can no longer identify where those parts are in space and how the body parts relate to each other. Therefore, aside from sensory and motor impairments, adults with SCI also experience mental body representation (MBR) deficits [5,6]. Body awareness and visuospatial body maps, both generated in the brain, are types of MBRs. Body awareness refers to an attentional focus on and awareness of internal body sensations, which includes whole-body awareness as well as awareness of body parts in relation to each other [7,8]. Visuospatial body maps help the brain understand where the body and specific body parts are situated in space. An example of altered visuospatial body maps can be empirically seen during the International Standards for Neurologic Classification of Spinal Cord Injury American Spinal Injury Association Impairment Scale neurologic examination [9], used in the clinic to quantify changes in sensory and motor function in adults with SCI; for example, people feel a touch with a cotton ball at the bottom of their foot as if it is on the top of their foot, or people feel a touch on the lower leg as being touched on their upper leg. In sum, deficits in body awareness and altered visuospatial body maps are referred to as MBR deficits. Body awareness and visuospatial body maps are situated in different parts of the brain—insular-parietal opercular cortex and posterior parietal cortex, respectively—which continuously interact with each other [6,10]. The visuospatial body maps are updated in real time at each moment to guide movements.

Whole-body awareness is developed by synthesizing information from all our senses. This integration occurs within the multimodal integration network of the brain, which includes regions such as the insula and parietal operculum [6,10]. The integration of senses involves not only perceiving which part of the body is being touched but also accurately determining the direction of auditory signals; for example, when we hear a car approaching, we instinctively look in that direction and wait for it to pass before crossing the street. To create a comprehensive understanding of whole-body awareness, the positions of body parts relative to other body parts, and our body position in space, the brain must be aware of the size, location, and dimensions of our body. Consequently, evaluating these aspects is crucial for identifying any potential MBR deficits a person may have [6,10-13].

Our previous research and that of others identified deficits related to MBR in adults with SCI [5,6,10,13-15]. MBR deficits are also thought to contribute to the production or maintenance of chronic neuropathic pain because the insula and parietal operculum are also key areas for chronic pain perception [5,14,16-19].

The Revised Body Awareness Rating Questionnaire (BARQ-R) [6,15,20,21] and the Multidimensional Assessment of Interoceptive Awareness-2 (MAIA-2) [22] were originally designed to address bodily awareness in adults with chronic pain and mindfulness in healthy adults, respectively. However, these scales do not identify awareness of body parts relative to other body parts, nor whole-body awareness; rather, they focus on interoceptive and emotional awareness, such as the person’s perception of tension and pain in their body, whether digestion impacts how they feel, or whether they show to others how they feel [6,15,20-22]. To the best of our knowledge, there is no measure available that assesses SCI-specific MBR deficits. In addition, psychometric assessment, such as interrater reliability, test-retest reliability, feasibility, utility, face validity, concurrent validity, and other relevant analyses, is important before using the scale in the clinic or for research.

Therefore, our overall aim is to develop an MBR assessment for individuals with SCI (ie, the SCI-BodyMap) and to assess its psychometric properties. We will also assess the SCI-BodyMap in uninjured adults (n=30) to identify whether healthy adults score statistically different on the scale than adults with SCI.

In addition, other factors may further influence MBR. Therefore, we will determine correlations between the SCI-BodyMap and age, time since SCI, levels of neuropathic pain, functional mobility level, and percentage of time spent using a wheelchair. In addition, we will conduct exploratory secondary analyses to determine whether there are significant differences in MBR deficits, assessed with the SCI-BodyMap, between adults with paraplegia versus those with quadriplegia, complete versus incomplete SCI, presence versus absence of spasms, and presence versus absence of current and past body awareness training.

### Purpose of This Study

The purpose of this study is twofold: (1) to develop the SCI-BodyMap for adults with SCI with neuropathic pain and those without neuropathic pain; and (2) assess the interrater reliability, test-retest reliability, concurrent validity, feasibility, utility, and face validity of the new SCI-BodyMap.

#### Aim 1

We will generate and refine items for the SCI-BodyMap for adults with SCI through iterations based on feedback from physical therapists (PTs) or occupational therapists (OTs) and adults with SCI.

#### Aim 2

Interrater reliability will be evaluated through in-person testing with the first author (SC) and 3 PTs not involved in initial item generation. For the self-reported items in the scale, test-retest reliability will be performed. Concurrent validity will be evaluated by correlating the SCI-BodyMap with the BARQ-R [13,20,21] and the MAIA-2 [22]. Feasibility, utility, and face validity will be assessed using the QQ-10 [23] after an in-person assessment of the scale with a PT or OT and an adult with SCI. We will also administer the SCI-BodyMap to uninjured adults (n=30) to identify whether healthy adults score statistically different on the scale than adults with SCI.

#### Secondary Analysis

We will also calculate correlations between the SCI-BodyMap and age, time since SCI, levels of neuropathic pain (the Numeric Pain Rating Scale [24]), functional mobility level, and percentage of wheelchair use. In addition, we will determine whether there are significant differences in MBR deficits, assessed with the SCI-BodyMap, between adults with paraplegia versus those with quadriplegia, complete versus incomplete SCI, presence versus absence of spasms, and presence versus absence of current and past body awareness training.

## Methods

### Overall Study Design

This study uses a cross-sectional observational design. Aim 1 will include item generation and refinement. Aim 2 will include interrater reliability, test-retest reliability, concurrent validity, feasibility, utility, and face validity analyses. Table 1 displays the study timeline.

**Table 1:** Scheduling of enrollment and assessments.

	Study period				
	Aim 1		Aim 2		
	Wk 1: enrollment+visit 1 (Zoom)	Wk 1: visit 2 (in person)	Wk 1: enrollment+visit 1 (Zoom)		Wk 2-3: visit 2 (in person)
Eligibility screen	✓		✓		
HIPAA^a^-compliant electronic consent	✓		✓		
Enrollment	✓		✓		
Demographic and clinical information	✓		✓		
Item-generation feedback survey	✓				
SCI^b^-BodyMap+in-person feedback		✓			✓
SCI-BodyMap in-person testing					✓
SCI-BodyMap self-report testing	✓	✓			✓
QQ-10 survey		✓			✓

^a^HIPAA: Health Insurance Portability and Accountability Act.

^b^SCI: spinal cord injury.

### Study Setting

Surveys will be distributed remotely through the University of Minnesota (UMN) REDCap (Research Electronic Data Capture; Vanderbilt University) platform. In-person visits will be conducted at the Brain Body Mind Lab at the UMN.

### Participants and Recruitment

For aim 1, we will recruit PTs or OTs who are board-certified neurologic clinical specialists with experience in working with adults with SCI or with experience in cognitive multisensory rehabilitation (CMR) [10,13]. We will recruit PTs and OTs through collaborations within the Minnesota Regional Spinal Cord Injury Model System and through collaborations with the Brain Body Mind Lab at the UMN.

For aim 1 and aim 2, we will recruit English-speaking adults with SCI, at least a year after SCI onset, with or without neuropathic pain. There is no restriction regarding race, ethnicity, age, level of injury, or completeness of injury. We will exclude adults with cognitive impairments, those who are pregnant, or those with deep pressure sores hindering prolonged sitting or lying down. For aim 2, we will also recruit English-speaking uninjured adults (aged ≥18 y). Uninjured adults and community-dwelling adults with SCI will be recruited through a contact list of participants identified via ClinicalTrials.gov, hospitals, StudyFinder, Research Match (a UMN registry for volunteers), and rehabilitation centers affiliated with the Minnesota Regional Spinal Cord Injury Model System, as well as individuals who have expressed interest in participating in research conducted at the Brain Body Mind Lab.

### Demographic and Clinical Outcomes

For healthy adults, we will gather demographic information such as age, sex assigned at birth, gender identity, race and ethnicity, and type and amount of current and past body awareness training (eg, martial arts, Pilates, yoga, tai chi, or qigong).

In addition, for PTs and OTs, we will collect professional information, including their title, profession, and credentials; whether they provide care for patients hospitalized in the clinic (inpatient) or whether these patients have appointments with them as outpatients; location of employment (eg, hospital and private practice); number of years working with adults with SCI; description of any certifications related to CMR (if applicable); and any other relevant information that they may want to add.

For adults with SCI, we will collect the same demographic information as well as SCI-specific information, including the cause of SCI; time since SCI (in months); completeness of injury (International Standards for Neurologic Classification of Spinal Cord Injury American Spinal Injury Association Impairment Scale grade [9]); level of SCI; highest, average, and lowest levels of neuropathic pain in the prior week (Numeric Pain Rating Scale [24]); location of pain; whether they have paraplegia, quadriplegia, or Brown-Séquard syndrome; spasm frequency and severity (Penn Spasm Frequency Scale [25]) and sensorimotor function (National Institute of Neurological Disorders and Stroke–Common Data Element Spinal Cord Injury Functional Index/Assistive Technology [26]), which addresses levels of difficulty in performing basic mobility, self-care, fine motor function, and ambulation; type and percentage of wheelchair or other ambulatory assistive device use; and, if applicable, type and amount of prior or current body awareness training and rehabilitation (eg, qigong or CMR).

### Item Generation and Refinement

**Figure 1:**
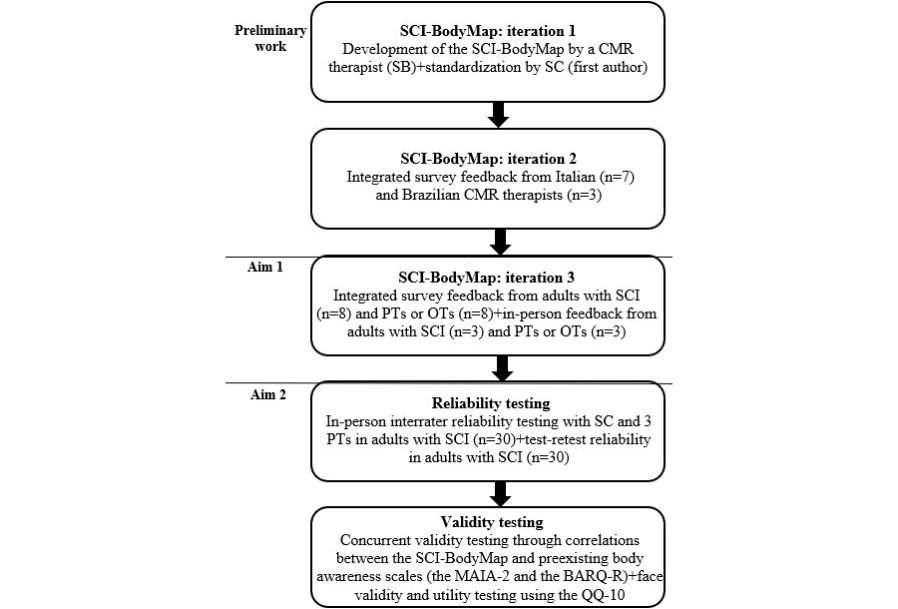
Study flowchart: item generation of the SCI-BodyMap. The flowchart illustrates the development process of the SCI-BodyMap. BARQ-R: Revised Body Awareness Rating Questionnaire; CMR: cognitive multisensory rehabilitation; MAIA-2: Multidimensional Assessment of Interoceptive Awareness-2; OT: occupational therapist; PT: physical therapist; SCI: spinal cord injury.

The study flowchart (Figure 1) shows the progression of the generation and refinement of items for the SCI-BodyMap. As preliminary work, an Italian PT specialized in CMR, MBR, and SCI will develop the initial items for the SCI-BodyMap (Figure 1: iteration 1). The first author (SC) will then standardize the items. Seven Italian PTs specialized in CMR and 3 Brazilian PTs specialized in CMR will jointly provide feedback so that the items can be refined (Figure 1: iteration 2).

Once feedback is integrated, 8 PTs or OTs who are board-certified neurologic clinical specialists and 8 adults with SCI will provide feedback on iteration 2 through a survey sent via email. Next, SC and 3 PTs will assess 3 adults with SCI with the SCI-BodyMap during an in-person visit (Figure 1: iteration 3), who will provide additional feedback. Decisions to retain, modify, or eliminate items can be made based on qualitative feedback from the CMR therapists, PTs, OTs, and adults with SCI in relation to the ease of use, feasibility of logistics, and clarity and delivery of instructions. We will incorporate feedback and propose solutions for conflicting suggestions to reach consensus.

### Scoring and Interpretation and Description of the SCI-BodyMap

The SCI-BodyMap will consist of 2 sections that will be scored and administered independently. The first section will include 4 items, which will be administered in person by a PT or OT to produce a total score. Scoring for these 4 items will be rated as incorrect or correct for each movement.

In item 1, participants will be instructed to estimate, without physically measuring, how many of their own hands would be required to measure specific body parts if they were to place their hands sequentially on these body parts. To answer accurately, participants need an intact body image and a clear visuospatial body map to understand both the size of their own hands and relate this to the dimensions of each body part.

In item 2, participants will sit with their feet flat on the floor. The PT will move the participants’ feet back and forth and then position one foot in a specific way. With their eyes closed, participants must determine the location of their foot in space, how this position relates to their other foot, and how it corresponds to an external object (eg, a marker placed on the floor in front of their feet). Answering this item accurately requires participants to have an understanding of their body’s position in relation to the environment and the positioning of both feet.

In item 3, the PT will hold the participant’s hand and slowly move it 6 to 8 inches above different body parts. With their eyes closed, participants must determine whether their hand would touch or miss a body part if their hand were to be lowered as well as which body part it would touch. Answering this item correctly requires participants to have a good sense of where their body is in space, an understanding of their body’s dimensions, and an awareness of how their hand interacts with other body parts.

In item 4, the PT will make snapping sounds at various locations around the participant’s body, approximately 2 feet away. With their eyes closed, participants must identify whether the sound is coming from the front or back of their body at head or foot level. In addition, with the snapping sound presented on the side of the body, they must determine whether the sound is at head, hip, or foot level. Answering this item correctly requires participants to have a strong awareness of their body’s position in relation to external auditory stimuli.

The second section contains 4 self-report items, scored separately. In item 1, participants will color the body parts on an image of a blank figure according to statements related to their perception of sensation, body awareness, and neuropathic pain. The score is based on the colored areas, sectioned into multiple body regions, corresponding to differing degrees of sensation (eg, numbness; ranging from 0=complete loss of sensation to 2=complete ability to sense touch and pressure), body awareness (ranging from 0=lack of bodily awareness or connection to the body to 2=complete bodily awareness and connection to the body), and neuropathic pain (ranging from 0=no neuropathic pain to 2=neuropathic pain perceived as stabbing, burning, or painful pins and needles).

For items 2, 3, and 4, scores are assigned for each body region based on the frequency of neuropathic pain (ranging from 0=no neuropathic pain to 4=neuropathic pain experienced 100% of the time), duration (ranging from 0=no neuropathic pain to 4=neuropathic pain experienced 100% of the time), average pain intensity (ranging from 0=no neuropathic pain to 4=excruciating neuropathic pain), and highest pain intensity (ranging from 0=no neuropathic pain to 4=excruciating neuropathic pain). The self-report section of the SCI-BodyMap focuses on how participants perceive their own bodies concerning sensations, pain, and body awareness, reflected here by their emotional or physical connectedness and awareness of their body.

The cutoff values and interpretation of scores for the SCI-BodyMap will be determined after assessing the scoring range in uninjured adults.

### Interrater Reliability Testing

To assess interrater reliability, SC (the first author) and 1 of the 3 PTs not involved in developing the SCI-BodyMap will administer the SCI-BodyMap to 30 adults with SCI [27]. In addition, SC will evaluate 30 uninjured individuals with the SCI-BodyMap.

### Feasibility, Utility, and Face Validity Testing

Upon completion of the SCI-BodyMap, each participant with SCI will fill out the QQ-10 [23]. This survey consists of 10 questions that assess the feasibility, utility, and face validity of the items and the scale as a whole. Respondents will rate their answers on a 5-point Likert scale ranging from strongly agree to strongly disagree. In addition, there are 3 open-ended questions regarding the relevance of the SCI-BodyMap, its ease of use, the time it takes to complete, and the emotions experienced during the process. We will incorporate qualitative data in the form of direct quotes in the manuscript reporting on the study results.

### Statistical Analyses

The sample calculation for aim 2 (n=30 participants for interrater and test-retest reliability) is based on the recommended sample size for pilot studies [27].

For both aims 1 and 2, we will summarize descriptive statistics for all demographic information collected. In aim 2, interrater reliability of the SCI-BodyMap (scores assigned by SC and 1 of the 3 PTs) will be evaluated with the Cohen κ coefficient and intraclass correlation coefficient (ICC). We will consider an ICC value of at least 0.75 as good interrater reliability and 0.90 as excellent interrater reliability [28]. We will consider the Cohen κ agreement of 0.60 as a moderate agreement and 0.80 as a strong agreement [29].

Test-retest reliability, also analyzed with the ICC, will be assessed within a 2-week period, a time frame commonly used in similar studies. Concurrent validity will be assessed using Spearman correlation coefficients between the SCI-BodyMap and the BARQ-R [21] and MAIA-2 [30]. A Spearman ρ correlation of 0.70 is a high correlation, and 0.90 is a very high correlation [31]. To assess feasibility, utility, and face validity, the QQ-10 Likert scale section will produce both “value” and “burden” scores ranging from 0 to 100, with higher “value” scores closer to 100 and lower “burden” scores closer to 0 indicating greater feasibility, utility, and face validity [23]. To be consistent with previous research, we will interpret mean “value” scores of at least 72.6 as high value and mean “burden” scores of ≤27.7 as low burden, indicating greater feasibility, utility, and face validity of the SCI-BodyMap [23,32]. We will also report responses to open-ended questions as direct quotes.

For our secondary analyses, we will determine associations between the SCI-BodyMap and age, time since SCI, levels of neuropathic pain, functional mobility level, and percentage of wheelchair use with Pearson or Spearman correlation coefficients, depending on the level of measurement and whether or not the data is normally distributed. In addition, we will conduct a Mann-Whitney *U* test to identify possible differences in MBR deficits, assessed with the SCI-BodyMap, between adults with paraplegia versus those with quadriplegia, complete versus incomplete SCI, presence versus absence of spasms, and presence versus absence of current and past body awareness training.

We will also perform a Mann-Whitney *U* test to evaluate whether there are significant differences in SCI-BodyMap scores in adults with SCI versus uninjured adults.

### Ethical Considerations

This study will be conducted in accordance with the ethical guidelines of the Declaration of Helsinki and was approved by the UMN institutional review board (IRB; STUDY00020888). The Health Insurance Portability and Accountability Act (HIPAA)–compliant REDCap electronic consent form is delivered via a secure survey link emailed to participants. We will take special precautions to protect confidentiality (ie, verify the participant’s email over the telephone, send a “test email,” and wait for an email reply before sending the HIPAA-compliant electronic consent form through REDCap).

The participants will have the same consent discussion with the researcher via telephone or Zoom (Zoom Video Communications, Inc) that they would have had in person (including the researcher asking questions to gauge comprehension and answering the participants’ questions).

The consent form will provide information regarding the study design, procedures, potential risks and benefits, alternatives to participation, voluntary nature of participation, and confidentiality. Participants will receive US $20 upon completion of the aim 1 email survey, an additional US $20 for the aim 1 visit, and an additional US $20 for the aim 2 visit. The first author (SC) will ensure participants’ understanding of the materials discussed and secure informed consent as well as ensure that they are aware of the voluntary nature of study participation and that they understand all potential benefits, risks, and alternatives to study participation as part of the informed consent process.

A PDF file of the HIPAA-compliant electronic consent form signed by both parties will be emailed to the participants for their records. The first author (SC) will document the consent process in a separate note, which will be stored in the UMN Box Secure Storage. Study procedures will not begin until all aspects of the remote consent process are complete. A copy of the HIPAA-compliant electronic consent form will not be included in the participant’s electronic health record, but the signed PDF file will be stored securely in the UMN REDCap platform and in the UMN Box Secure Storage. REDCap uses a MySQL database via a secure web interface, with data checks to ensure data quality during data entry. All UMN IRB and HIPAA rules will be followed.

The principal investigator (AVdW) will oversee the safety of the study at the UMN site. We anticipate minimal risk, given that the assessments conducted with the SCI-BodyMap are similar to those conducted in the clinic or rehabilitation centers. The questionnaires will be completed over the secure UMN Zoom system. The only risk here would be the sensitivity of some questions related to SCI-related symptoms. There are minimal potential risks associated with data privacy and confidentiality, and care will be taken to avoid them, for example, by setting up a waiting room for the Zoom meetings. All data are stored on HIPAA-secure servers (REDCap and UMN Box Secure Storage). Although adverse events are unlikely, given the nature of the study, all participants will be encouraged to contact the principal investigator or study staff if they experience any side effects or adverse events. In such cases, applicable policies and procedures of the UMN IRB will be followed.

## Results

At the time of manuscript submission, the study is being conducted under the approved UMN IRB protocol version 3.0. We do not anticipate further changes. Recruitment started on November 18, 2024, and we estimate that we will complete data collection by the end of September 2025. As of August 2025, we have enrolled 16 participants (PTs or OTs: n=8, 50%; adults with SCI: n=8, 50%) for aim 1 and 42 participants (adults with SCI: n=29, 69%; adults without SCI: n=13, 31%) for aim 2. The results of this study will be reported and submitted as a manuscript to scientific journals as well as disseminated through presentation at national and international conferences in the fields of SCI and complementary and integrative medicine.

## Discussion

### Summary

This project aims to develop a new scale to measure MBR deficits, called the SCI-BodyMap. The items on this scale will be designed with, and tailored to the experience of, adults with SCI. We expect the SCI-BodyMap to demonstrate good to excellent interrater reliability, test-retest reliability, and concurrent validity.

Previous studies have used body awareness scales to assess MBR in adults with SCI. However, they are no scales specifically designed to address MBR deficits in adults with SCI [33,34]. Some of the existing body awareness or pain scales share similarities with our self-report measure, such as aspects related to coloring [35]; statements addressing feelings of disconnectedness or self-perception of awareness [36]; or tasks related to the visuospatial map, such as the Body Image Task [33]. Nevertheless, there is currently no scale that specifically targets both types of MBRs (eg, body awareness and visuospatial map) in individuals with SCI. Moreover, the SCI-BodyMap includes both an objective section administered by PTs or OTs and a self-report section.

### Limitations

Given the small sample size, meaningful associations for some of our secondary analyses may be difficult to detect, limiting generalizability.

### Future Directions

The next psychometric steps for the SCI-BodyMap are assessing reliability, validity, and responsiveness to change and calculating the minimal clinically important difference in a larger sample of adults with SCI. Given that this scale will be validated in English, future studies should aim to validate the scale in different languages.

## Abbreviations

BARQ-R: Revised Body Awareness Rating Questionnaire
CMR: cognitive multisensory rehabilitation
HIPAA: Health Insurance Portability and Accountability Act
ICC: intraclass correlation coefficient
IRB: institutional review board
MAIA-2: Multidimensional Assessment of Interoceptive Awareness-2
MBR: mental body representation
OT: occupational therapist
PT: physical therapist
REDCap: Research Electronic Data Capture
SCI: spinal cord injury
UMN: University of Minnesota
